# Comprehensive improvement of nutrients and volatile compounds of black/purple rice by extrusion-puffing technology

**DOI:** 10.3389/fnut.2023.1248501

**Published:** 2023-10-11

**Authors:** Yanrong Ma, Jiaxing Li, Yan Xue, Yunbi Xu, Chunming Liu, Dingding Su

**Affiliations:** ^1^Peking University Institute of Advanced Agricultural Sciences, Weifang, China; ^2^School of Advanced Agricultural Sciences, Peking University, Beijing, China

**Keywords:** black/purple rice, extrusion puffing technology, metabolomics, flavonoids, volatile compounds

## Abstract

**Introduction:**

Black/purple rice is a pigmented rice variety that contains high levels of anthocyanins, flavonoids, and other valuable bioactive compounds. Owing to its robust anti-inflammatory and antioxidant properties, black/purple rice exerts a beneficial effect on human health. Extrusion puffing technology has emerged as a promising means of improving rice flavor with lesser effect on nutrient content. In this study, metabolomics approach was used to conduct comprehensive metabolomics analyses aimed at examining the impact of extrusion puffing on black/purple rice nutritional value and flavor.

**Methods:**

Firstly, the basic nutrient composition contents and extrudate characteristics of black/purple rice and Extrusion puffed black/purple rice were conducted. Then metabolomics profiling analyses of black/purple rice samples were performed to explore the impact of the extrusion puffing process on nutrient content and bioactive properties, in which we quantitatively determined the flavonoids and evaluated relative contents of volatile compounds.

**Results:**

These analyses revealed that following extrusion puffing, black/purple rice exhibited significant improvements in the content of nutrients including flavonoids, minerals, and proteins together. Extrusion puffing additionally increased the diversity of volatile compounds within black/purple rice.

**Discussion:**

These results suggest that extrusion puffing represents an effective means of substantially improving the functional and nutritional properties of black/purple rice, offering beneficial effects on consumer health. Overall, these data provide novel insights into the quality of extrusion puffed black/purple rice that will guide future efforts to establish how extrusion puffing can alter the nutrient content in a range of foods, thereby supporting the further development of a range of healthy food products.

## Introduction

Rice (*Oryza sativa* L.) is a staple crop throughout the world that is rich in minerals, vitamins, fiber, protein, unsaturated fatty acids, polysaccharides, and flavonoids found in the pericarp, seed coat, aleurone, germ, and endosperm. Historically, however, culinary preferences have favored polished rice, which exhibits a ~ 80% drop in nutrient content relative to bran (1). Therefore, brown rice that retains the outer bran layer has grown somewhat in popularity in recent years owing to their higher levels of anti-inflammatory and antioxidant compounds as compared to those present in common white rice (2, 3). Compounds present in these rice varieties include alkaloids, coumarins, and flavonoids, the latter of which are polyphenolic secondary metabolites produced by a wide range of vegetables, fruits, and other crops (3). Flavonoids are widely considered to exert a range of beneficial anti-inflammatory, anti-aging, cardioprotective, and anti-atherosclerotic activities. Flavonoid content in rice is closely associated with rice color, and there are marked differences in such content among varieties of rice (4). Currently, black/purple rice is mainly used as the main ingredient of porridge, beverages, and bread on the market (5). Overall, black/purple rice is a kind of characteristic agri-product resource that has not been fully used, and its development potential is huge. Therefore, the modern food processing technology is applied to black/purple rice processing to reduce the loss of functional components in the processing of black/purple rice according to research results of structure, property, content, and mechanism of various functional factors in black rice. Development of black/purple rice-base health food with definite functional role will produce enormous social and economic benefits.

Extrusion puffing technology is a well-known technique that can be able to produce a diverse range of food products. This technology boasts the advantages of diversified products, continuous and low labor-intensive production, superior yield, and negligible environmental impact (6). The thermomechanical extrusion puffing process entails the brief exposure of particular samples to high levels of temperature, pressure, and shear force, resulting in changes of covalent bonds and physical structures of macromolecules (7). The raw materials are poured into the puffing extruder, then transported to the inner temperature control area for rapid cooking before being extruded through a die. Pressure drops, extrudates exhibit loose and porous appearances, accompanied by crispy textures that delight the senses. Alongside the aforementioned structural changes, the extrusion puffing process induces notable transformations in the constituent ingredients of raw materials, including starch gelatinization, protein denaturation and recombination, fiber degradation, and pathogen elimination. As reported, this technique can enhance the content of soluble dietary fiber content, phenolic levels, digestibility, and nutrient bioavailability within processed foods (8), which generate widespread interest in its application toward the manufacture of breakfast cereals, precooked grains, and other cereal-based food products. Carmo et al. (7) utilized extrusion technology to develop an expanded snack product enriched with β-glucan from a mixture comprised of pea starch, pea protein, and oat fiber-rich fractions. Chien et al. (6) used certain high-starch cereals such as corn, brown rice, and buckwheat to produce high-maltose syrup through extrusion puffing. Zapana et al. (9) investigated the effect of extrusion puffing on starch-chitosan coating of puffed quinoa, discovering that the technology can produce high-quality popped quinoa characterized by enhanced biological availability of organic matter. Generally, extrusion puffing technology is widely applied to expand food resources and improve food nutrition and taste. In recent years, in terms of nutrition analysis, previous studies have primarily focused on changes in the total phenols and total flavonoids content of cereals during the process of extrusion puffing. There have been few publications about the alterations in the individual compounds profiles of phenols or flavonoids using metabolomics approach during extrusion puffing remain unexplored (8, 10). Notably, the influence of extrusion puffing on individual flavonoid compounds of black/purple rice has yet to be reported.

Metabolomics is an emerging analytical tool utilized to investigate the final products of gene expression with a low molecular weight (<1,000 Da) in living organisms (10). Based on the different research purposes and methods, metabolomics is generally classified into non-targeted metabolomics and targeted metabolomics analyses. The former has relatively simple operation but lower sensitivity, which makes it challenging to accurately identify related metabolites, and necessitates dependence on the public database for identification. In contrast, targeted metabolomics offers a higher sensitivity and enables absolute qualitative and quantitative analysis of a limited number of target metabolites, which is helpful to further investigating the specific regulation mechanism (11). In recent years, metabolomics has been increasingly applied to various fields such as food science (10), medical science (12), plant growth and development (13) and others (14, 15). Therefore, we applied this method to qualitatively and quantitatively analyze flavonoids as well as qualitatively analyze volatile metabolites and their changes in black/purple rice after extrusion puffing, aiming to reveal the formation mechanism of the nutrition and flavor of black/purple rice.

Considering flavonoids, volatile metabolites, and their changes during extrusion puffing, ultra-performance liquid chromatography with a tandem mass spectrometry (UPLC-MS/MS) and gas chromatography-ion mass spectrometry (GC-IMS) was employed to identify metabolites, respectively. The significantly different metabolites were identified to clarify the impact of extrusion puffing on the enhancement of the nutrition, flavor, and antioxidant activities of black/purple rice. As such, these findings provide a robust theoretical foundation for further efforts to apply extrusion puffing as a means of preparing healthy food.

## Materials and methods

### Materials

The Zhongzi 4 variety of black/purple rice (designated as BR) was obtained from Peking University and used as the experimental focus in this study.

### Extrusion

Extrusion puffed black/purple rice (EBR) was generated using an SLG40-A twin-screw extruder (Dayi Machinery Co., Ltd., Tsinan China) (Supplementary Figure S1), the barrel of which was separated into four temperature-controlled zones (65°C, 100°C, 130°C, and 155°C). Extrusion parameters were as follows: screw diameter = 70 mm, screw length = 1,699 mm, length-to-diameter ratio = 24.27:1, die diameter = 3 mm, extruder screw speed = 900 rpm, feeding speed = 200 kg/h. Before extrusion, the fine flour was acquired by milling in a grinder (Sujata, India) and sieved (80 mesh). Moisture contents of raw material varied from 12 to 14%. After sifting, the sample was homogenized with deionized water to the moisture of 20%. A grinder (Sujata, India) was used to mill samples into a flower that was able to fully pass through an 80-mesh sieve for further analyses.

### Nutrient composition analyses

Moisture, protein, fat, and carbohydrate levels were assessed as per the respective AACC methods 44-15A, 46-11A, 30–10.01, and 12–21. AACC method 40–71.01 was used to determine levels of minerals (calcium, iron, zinc, and copper). All analyses were repeated in triplicate.

### Analyses of extrudate characteristics

#### Bulk density

The bulk density (BD) was assessed as reported previously by Hashemi et al. (16). BD was measured in a 500 mL graduated cylinder. BD was measured by volumetric replacement with hulled millet:


BDgcm3=MV

Where M is mass (g) and V is the volume of the beaker in cm^3^. In total, the BD of sample was expressed as the average of 3 determinations.

#### Water absorption index (WAI) and water solubility index (WSI) analyses

Using an approach detailed previously by Yang et al. (17), a 1.6–2.0 g sample (dry basis weight, W_0_) was mixed with 25 mL of dH_2_O in a centrifuge tube (W_1_), shaking until fully dispersed. This solution was then incubated for 30 min at 30°C in a water bath, stirring gently every 10 min. Samples were then spun for 15 min at 5,180 × *g* after which supernatants were transferred to a beaker that had been weighed (W_2_) and heated at 105°C until reaching a constant weight (W_3_). Precipitates and tubes were then weighed at the same time (W_4_). Testing was performed in triplicate.

Relative WAI and WSI proportions were determined with the following equations:


$$WAI=\frac{W_{4}-W_{1}}{W_{0}}$$

$$WSI\;\left(\%\right)=\frac{W_{3}-W_{2}}{W_{0}}\times 100\%$$

#### Color analyses

A CR-400 Chroma Meter (Konica Minolta, Japanese) with a measuring area 8 mm in diameter and a 0° viewing angle was used for the measuring of sample flour color. Prior to use, instrument calibration (Y = 93.7, x = 0.3135, y = 0.3199) was performed using a white calibration plate (CR-A43, Konica Minolta). Four color parameters were analyzed including L* (0 = black, 100 = white), a* (−a* indicates greenness and + a* indicates redness), b* (−b* indicates blue and + b* indicates yellow) and the total color difference (△E= $\surd {\left(\Delta \;a^{*}\right)}^{2}+{\left(\Delta \;b^{*}\right)}^{2}+{\left(\Delta \;L^{*}\right)}^{2}$ ). Sample measurements were performed 10 times.

### Qualitative and quantitative analysis of flavonoids metabolites

#### Sample preparation and extraction

Freeze-dried BR and EBR were ground into powder (30 Hz, 1.5 min) using a Grinding mill (MM 400; Retsch, Haan, Germany). The sample was stored at –80°C before further analysis. The sample powder (20 mg) was weighed and dissolved in 0.5 mL methanol (70%, v/v) and 0.01 mL internal standard solution (4,000 nmol/L). The extract was sonicated for 30 min and centrifuged at 12, 000 × *g* under 4°C for 5 min. The supernatant was filtered through a 0.22 μm membrane filter for further Ultra Performance Liquid Chromatography-Mass spectrometer (UPLC-MS/MS) analysis.

#### UPLC-MS/MS

The extraction of flavonoids metabolites in two samples was conducted by using a UPLC-MS/MS referring to the established method with minor modifications (18). The flavonoids metabolites were separated by a Waters ACQUITY UPLC HSS T3 C18 (2.1 mm × 100 mm, 1.7 μm) with an UPLC, with ultrapure water with 0.05% formic acid (A) and acetonitrile with 0.05% formic acid (B) as mobile phase. The elution gradient for flavonoids was from 10 to 20% of B for 1 min, and flavonoids elution linear gradient for B solvent was from 20 to 70% for 8 min, from 70 to 95% for 3.5 min and then reached to 95% at 12.5 min and held for 1 min, 95 to 10% for 0.1 min, and reached to 10% at 15 min. During elution, the column temperature was maintained at 40°C, and the injection volume was 2 μL. The flow rate was set at 0.35 mL/min.

The identification and quantification of flavonoids metabolites were operating in positive and negative ion mode and controlled by Analyst 1.6.3 software (Sciex). Temperatures of the Electrospray Ionization (ESI) was set as 550°C; the spray voltage was 5,500 kV for positive ion and the spray voltage was −4,500 kV for negative ion. Curtain gas (CUR) was set at 35 psi.

#### Data analysis

The mass spectrometric data were processed in Analyst 1.6.3 and MultiQuant 3.0.3 software. According to the retention time and peak type information of the standards, the chromatographic peaks detected in different samples were corrected for integration to ensure the accuracy of qualitative and quantitative analysis. The actual metabolite contents were calculated using the peak areas obtained and brought into the calibration curve.

Unsupervised principal component analysis (PCA) was performed by statistics function prcomp within R. The data was unit variance scaled before unsupervised PCA. Significantly regulated metabolites between groups were determined by variable importance projection (VIP) and absolute Log_2_FC (fold change). VIP values were extracted from orthogonal partial least squares discriminant analysis (OPLS-DA) result, which also contain score plots and volcano plots. The data was log transform (log_2_) and mean centering before OPLS-DA. The hierarchical cluster analysis (HCA) results of differential metabolites were present as heatmaps with dendrograms.

### GC-IMS

A GC-IMS (FlavourSpec®) instrument from the G.A.S. Department of Shandong Haineng Scientific Instrument Co., Ltd. was used for analyses of volatile compounds (VCs) using a slightly modified version of a previously reported protocol (19). Briefly, 5.0 g samples were placed in 20 mL headspace bottles and inserted into the sampling tank of an autosampler, followed by sample incubation for 15 min at 80°C. A 500 μL headspace volume was transferred automatically into the injector at 45°C using a warm (65°C) syringe. Nitrogen was used to transport the headspace into a chromatography column (MXT-5, 15 m × 0.53 mm), and mixed compounds were introduced into the drift region via shutter grid using the drift gas and electric field, with IMS detector monitoring. VCs were identified through comparisons of NIST and drift time, corresponding to the time (in ms) needed for ions to reach the collector through drift tubes, for standard compounds included in the IMS library. Samples remained intact throughout detection, and were analyzed in triplicate.

### Statistical analysis

Data are means ± standard deviation. Student’s t-tests followed by Duncan’s multiple range test were used to detect significant differences (*p* < 0.05) between the composition of BR and EBR samples. The 2022 Origin software (Stat-Ease Inc., v 8.0 USA) was used for figure generation.

## Results

### Physical properties analyses

**Figure 1:**
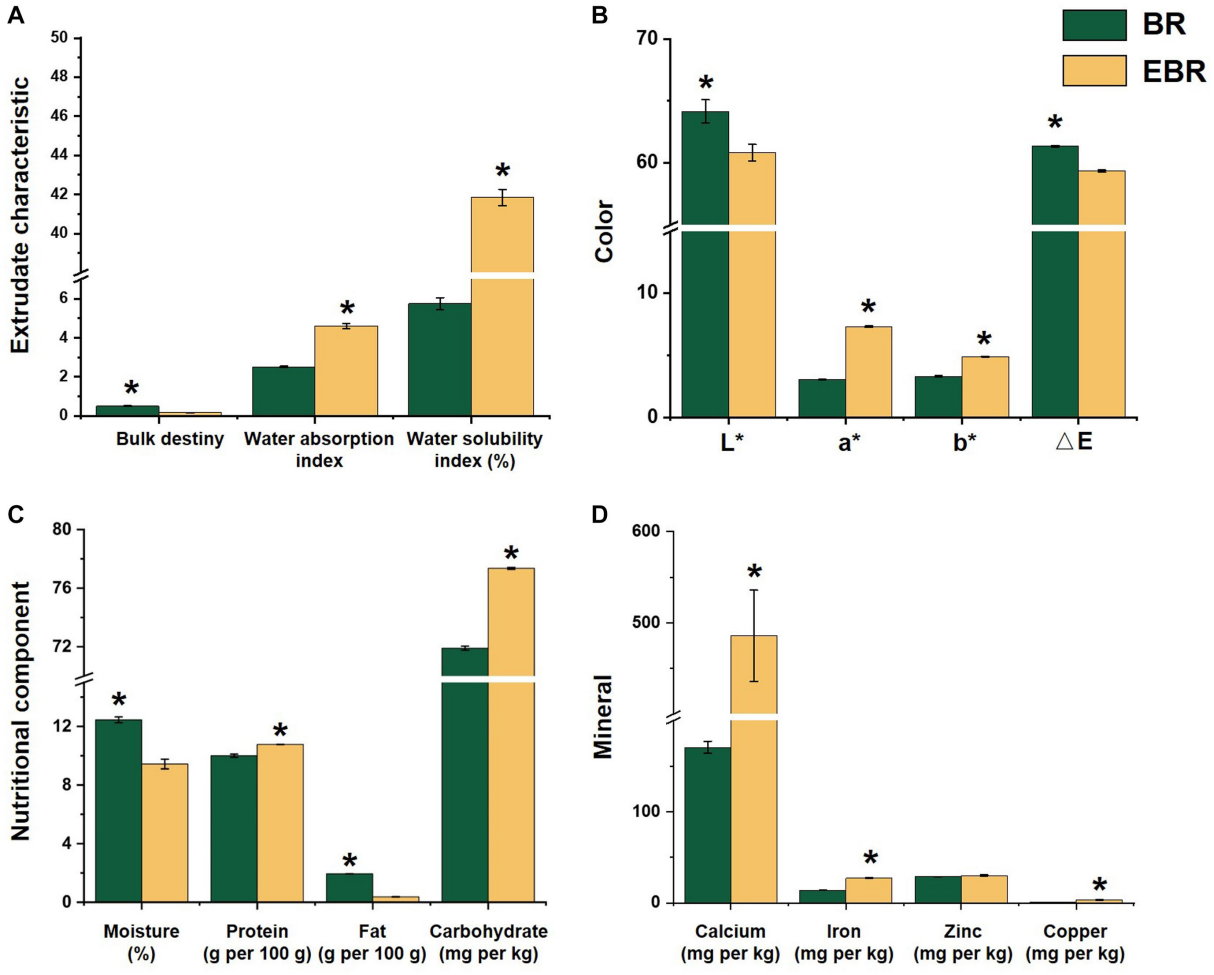
Physical properties and basic nutritional compositions and of BR and EBR. **(A)** Radial expansion ratio, water absorption index and water solubility index of two samples. **(B)** Color of two samples. **(C)** Moisture, protein, fat, and carbohydrate contents of two samples. **(D)** Four mineral (calcium, iron, zinc and copper) contents of two samples. *Indicates significant differences (*p* < 0.05).

The BD, which corresponds to the volumetric displacement of a given material following extrusion puffing, was next analyzed for EBR samples (Figure 1A), revealing a EBR bulk density of 0.168, which was 2.03 times lower than that before extrusion. WAI serves as a metric that represents the water retention capacity of starch following swelling in an excess of water, and it is associated with the degree of starch gelatinization. WSI measures the degradation of starch molecules during the extrusion process, increasing soluble polysaccharide levels therein (17). Relative to BR samples, EBR samples exhibited significant increases in WSI and WAI by 0.83 and 6.28 fold, respectively (Figure 1A), consistent with enhanced water solubility and rehydration of these extrudates following the extrusion puffing process. In terms of color, Relative to BR samples, the L* value of EBR samples fell from 64.15 to 60.81 and the △E value fell from 61.34 to 59.33, whereas the a* value rose from 3.05 to 7.32 and the b* value rose from 3.30 to 4.90 (Figure 1B), consistent with a decrease in brightness and corresponding increases in redness and yellowness. This aligns well with findings from a prior study in which rice bran was added to ready-to-eat extruded corn snacks (20).

### Basic nutritional composition analyses

The nutritional properties of BR and EBR samples were initially compared by evaluating basic nutritional characteristics such as fat, protein, moisture, carbohydrate, mineral, and vitamin content (Figure 1). As expected, the moisture content in BR (12.45%) was significantly higher than that in EBR (9.43%) (Figure 1C), thus enabling extrusion puffing to substantially prolong the shelf life of black/purple rice (9). Relative to BR, protein, and carbohydrate levels in EBR rose significantly. Notably, the fat content in EBR samples was lower than that in BR samples. These results align well with findings from a prior analysis of single-screw extruded fish and rice flour (21). Of the seven minerals analyzed in these samples, significant increases in iron and calcium content were evident in EBR relative to BR (Figure 1D), in line with a prior report (2). While no significant increases in potassium, magnesium, manganese, zinc, or copper were evident in EBR, these levels were still notably higher than those in many other staple foods such as wheat or white rice (22).

### UPLC-MS/MS-based identification of flavonoid profiles

**Figure 2:**
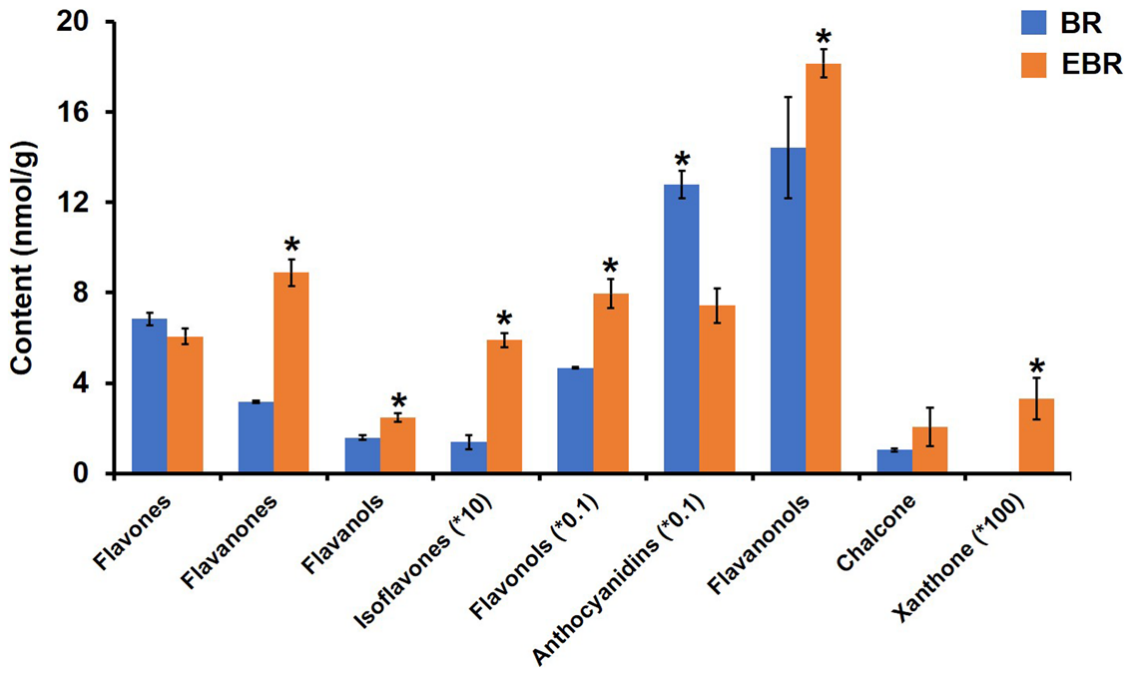
Nine kind of flavonoids (flavones, flavanones, flavanols, isoflavones, flavonols, anthocyanidins, flavanonols, chalcone and xanthone) contents of BR and EBR. *Indicates significant differences (*p* < 0.05).

The content and variety of flavonoids in rice are closely related to its color, and there are significant differences between different varieties. As shown Figure 2, the contents of the flavonoids change significantly in the process of extrusion puffing. Except for contents of flavones and chalcone had no significant difference, significant increases in the abundance of flavanones, flavanols, isoflavones, flavonols, and flavanonols were evident in EBR samples relative to BR, while the anthocyanidins content in black/purple rice decreased significantly after extrusion puffing (Supplementary Table S1). Strikingly, xanthones were detected in EBR samples yet were absent in BR samples, mainly including mangiferin and isomangiferin.

**Figure 3:**
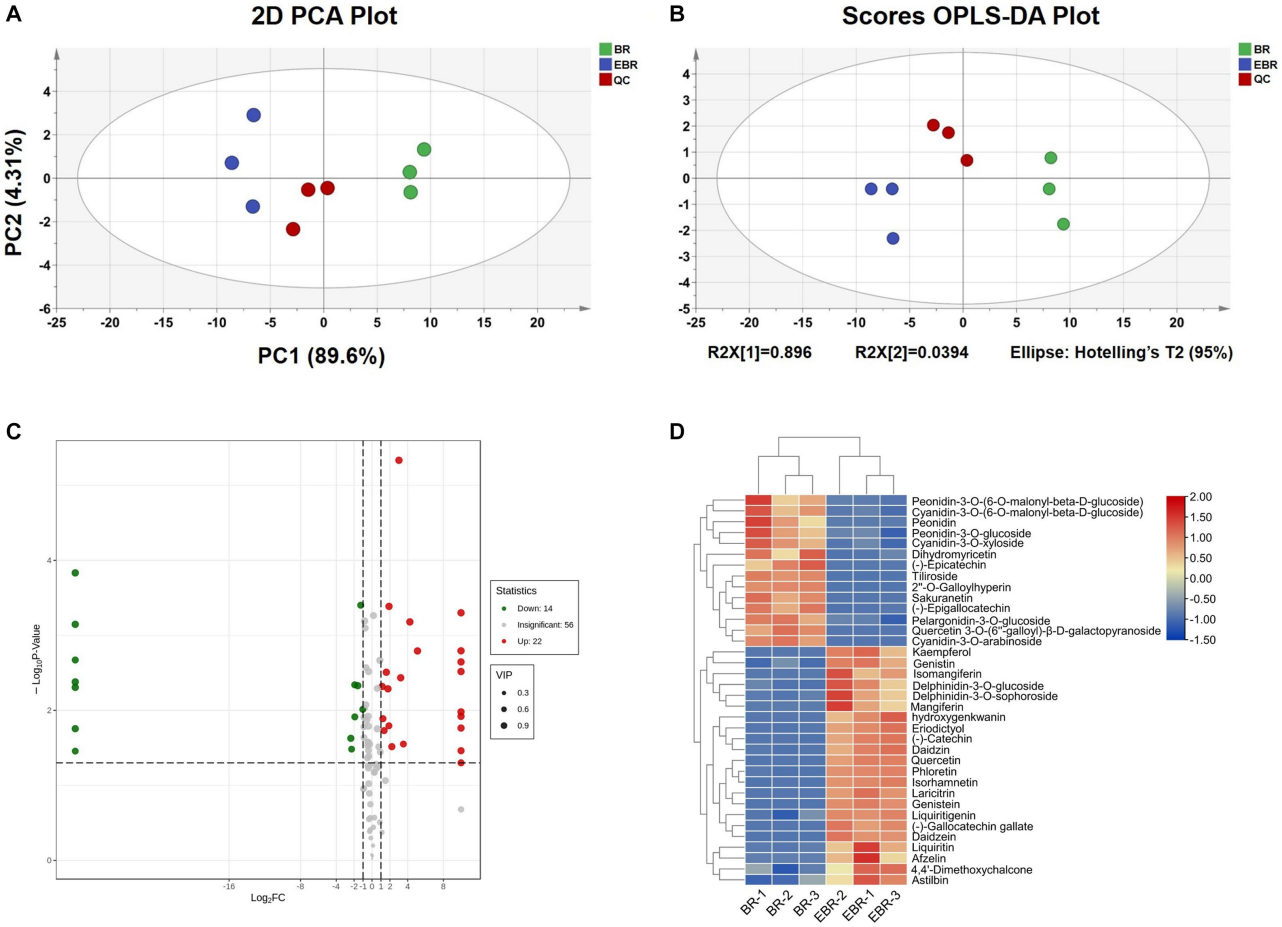
Metabolic profiles of flavonoids in BR and EBR. **(A)** PCA score plot of two samples. **(B)** Score plots generated from OPLS-DA in two samples. **(C)** The volcano plot showing the differential metabolites expression levels between BR and EBR. **(D)** HCA map of differential metabolites. Each sample is represented by a column, and each differential metabolite is displayed in a row.

A multivariate statistical analysis was performed for two rice groups to show the differences in their metabolites. A PCA plot was generated that highlights differences in flavonoid composition both within and between sample groups (Figure 3A), with PC1 and PC2, respectively, accounting for 61.40 and 14.08% of the overall variance (total: 75.48%). Samples were clearly grouped into distinct clusters highlighting differences in flavonoid content in BR and EBR in these plots. OLPS-DA models can be leveraged to filter out orthogonal variables not relevant to metabolite classification as a means of maximizing the differentiation between groups and identifying differentially abundant metabolites (3). BR and EBR samples were clearly separated in established OPLS-DA models (Figure 3B).

Based on the results of the OPLS-DA model, differential metabolite screening was next performed for all annotated metabolites in an effort to more fully clarify the differences in flavonoid content in BR and EBR samples (Figure 3C). In total, 36 metabolites were significantly (FC ≥ 2 or ≤ 0.5; VIP ≥ 1) differentially abundant, including 14 that were downregulated (flavanonols, flavones, flavonols, flavanols, and anthocyanidins) and 22 that were upregulated (chalcone, flavanones, flavanonols, anthocyanidins, flavones, flavonols, flavanols, xanthones, and isoflavanones). Among the downregulated metabolites, there are mainly seven anthocyanins, which are Peonidin-3-O-(6-O-malonyl-beta-D-glucoside), peonidin, peonidin-3-O-glucoside, cyanidin-3-O-arabinoside, cyanidin-3-O-xyloside, cyanidin-3-O-(6-O-malonyl-beta-D-glucoside), and pelargonidin-3-O-glucoside. On the other hand, the upregulated metabolites include 9 kinds of flavonoids, of which flavonols are the most upregulated among the differential metabolites, including quercetin, isorhamnetin, kaempferol, laricitrin and afzelin (Figure 3D).

### GC-IMS-based identification of volatile flavor compounds

**Figure 4:**
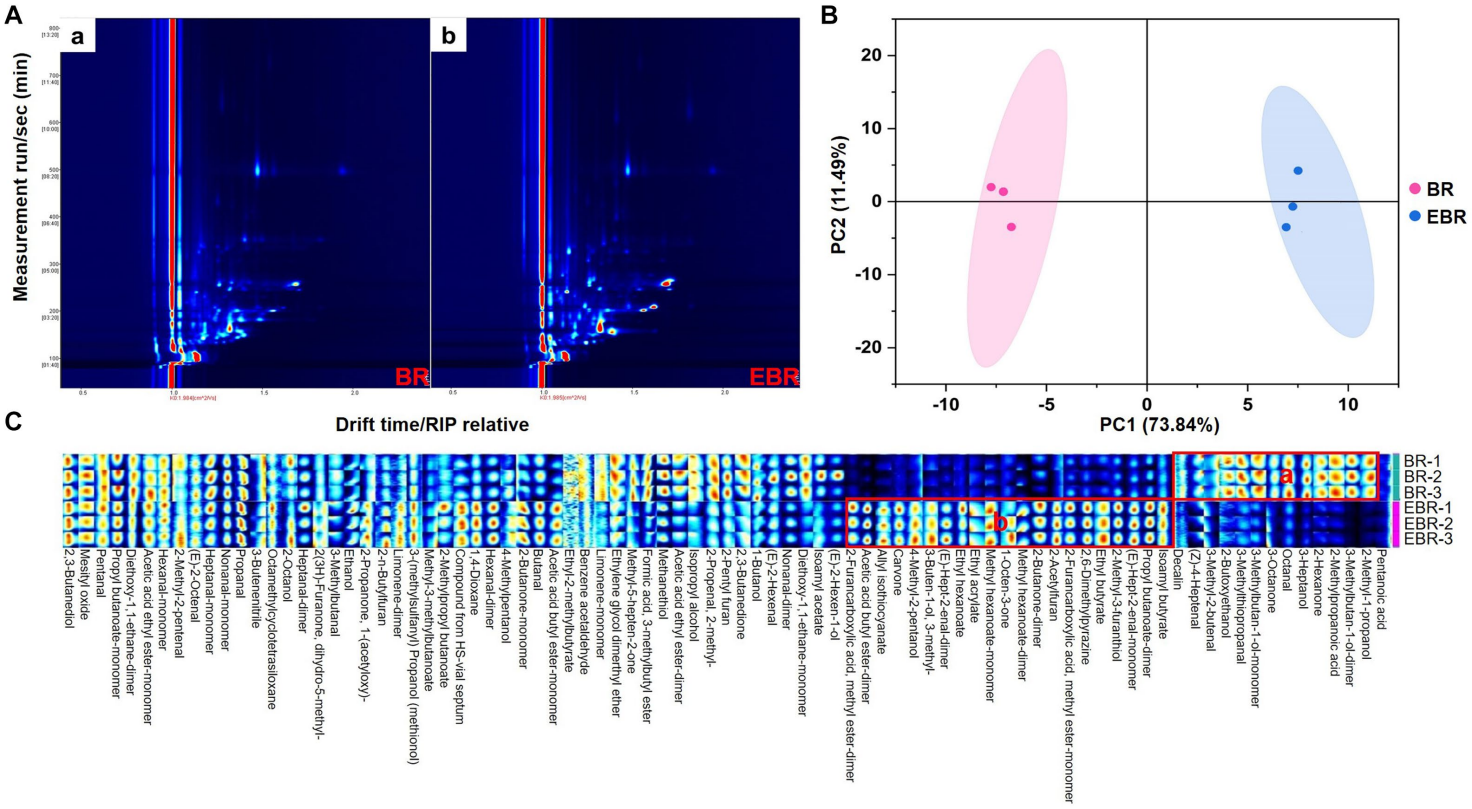
Volatile flavor compounds (VCs) of BR and EBR by GC-IMS. **(A)** GC-IMS topview plot of VCs in two samples. The background of the entire map is blue, and the red vertical line at abscissa 1.0 is the reaction ion peak (RIP, normalized). Each point on the right side of the RIP peak indicates a type of volatile compound (VC) found in the samples. The color represents the substance concentration, white indicates a lower concentration, red indicates a higher concentration and darker colors indicate the higher concentration. **(B)** PCA score plot. **(C)** Fingerprints of the VCs in two samples.

Flavor, like nutritional content, serves as a key index by which rice quality can be established, in addition to being closely tied to consumer preference. Volatile compounds (VCs) in BR and EBR samples were thus evaluated via GC-IMS (Figure 4). Relative to BR samples (Figure 4Aa), EBR samples were more scattered with several red dots (Figure 4Ab, arrows) consistent with higher VCs concentrations therein, A PCA analyses revealed clear differences in the VC content in these BR and EBR samples. We performed PCA in order to show the differences of the VCs in BR and EBR, with PC1 (73.84%) and PC2 (11.49%) accounting for 85.33% of overall variance, highlighting key differences between these samples (Figure 4B). The unique VCs fingerprint of each sample was additionally examined, providing an opportunity for the direct identification of VCs differentially abundant in these two sample types. A gallery plot was prepared in which rows and columns, respectively, represent samples and VCs (Figure 4C). The 85 detected peaks (P), 72 volatile components and 13 dimers were identified (Supplementary Table S2), including esters (15, 20 P), aldehydes (18, 23 P), ketones (9, 10 P), alcohols (14, 15 P), organic acids (2, 2 P), heterocyclics (7, 7 P), and other VCs (7, 8 P). Of the differentially abundant VCs identified herein, 5 alcohols, 4 aldehydes, 2 ketones, 1 organic acid, and 1 other VC (decalin) were detected in BR samples whereas EBR samples contained 11 esters, 3 ketones, 3 heterocyclics, 2 aldehydes, and 2 alcohols. While the levels of some aldehydes and alcohols declined following extrusion puffing, the ester, heterocyclic, and ketone content in these black/purple rice samples rose following processing.

## Discussion

Extrusion puffing technology is one of the most widely used processing methods in the food industry due to its low processing cost compared to other cooking and processing methods (23). At the same time, black/purple rice is typically a better source of important phytochemicals, fiber, and minerals and has gained popularity due to various active ingredients that prevents most of the chronic diseases (2). At high extrusion temperatures, the raw materials undergo changes in proximate composition including protein, fat, moisture, and carbohydrates, and the active substances such as flavonoids also undergo structural changes (21). However, the changes have not been studied comprehensively. Therefore, physical, nutritional properties of extrusion puffing black/purple rice were investigated and the changes in the profiles of individual compounds in the flavonoids by using metabolomics technology.

### Extrusion puffing enhanced nutritional values of black/purple rice

Extrusion puffing process is a highly-promising technique for the production of the rice and rice-based products, which greatly impacts the physicochemical properties of rice extrudates (2). In terms of physical properties of extrusion puffing, it has observed that the extrusion puffed sample has relatively lower radial expansion ratio (RER) value than that reported previously (23), which can be attributed to the higher screw rate accelerates the gelatinization of starch and may produce a higher pressure drop at the outlet of the die, thus promoting cell nucleation and expansion (16). Extrusion puffing led to black/purple rice have higher water absorption index (WAI) and water solubility index (WSI) values. Similar results were found by Zapana et al. (9) when examining the effects of extrusion puffing on the WSI and WAI properties of quinoa. During the process, the high pressure and temperature condition cause starch gelatinization and increase of the soluble substance levels, while the intrinsic starch structure undergoes damage, leading to higher WAI and WSI values (24). The loose and porous properties of the resultant extrudate thereby contribute to observed increases in WAI.

In terms of the basic nutritional compositions of rice, starch and protein are the primary components, and their contents increased significantly after processing. While proteins are generally heat-labile and profoundly affected by the extrusion-mediated cooking process (2), the degree of the changes in the nutritional characteristics of extruded products largely depends on both extrusion parameters and the raw materials (25). For instance, relative to exposure to 120°C temperatures, significant increases in the degradation of alkali-soluble proteins have been observed when extrusion temperatures are increased to 135°C or 150°C (2). Rice extrusion at temperatures ranging from 120 to150°C results in a marked drop in the gel viscosity of the resultant rice flour without decreasing the amylose content therein (26). A previous study also found that extrusion process could have a marked effect in changing some physicochemical properties of oat bran soluble dietary fiber and significantly increased its content due to the change of oat bran cell walls after the processing (27). Additionally, the decrease in fat content during extrusion puffing may be attributable to the high temperatures used, which can cause oxidation and degradation fatty acids (2). During food matrix processing, the minerals determined in this study have been found to remain stable when exposed to heat (28), and the similar result was also documented by Silva et al. (25), which brown rice and corn flour resulted in significant improvement in mineral content of pasta produced through this approach. The reasons for these results may be attributed to some anti-nutritional substances such as phytates and condensed tannins can form insoluble complexes with mineral ions, while extrusion puffing can remove these substances, thereby enhancing the availability and absorption of minerals (29). Given human cannot synthesize minerals including iron, calcium, zinc, and magnesium themselves, mineral-rich foods represent an ideal option for consumers (30). As such, the use of extrusion puffed black/purple rice may offer value for future use as a mineral-fortified food product.

### Extrusion-puffing improved content and species of flavonoids in black/purple rice

Bioactive compounds like flavonoids, alkaloids, and phenolic acid are the focus of growing interest due to their reported anti-inflammatory, antioxidant, and anticancer properties (4, 8). Higher flavonoid levels have previously been reported in pigmented rice (3). As such, a UPLC-MS/MS approach was next used to qualitatively and quantitatively assess the flavonoid profiles in these BR and EBR samples. According to the type, quantity and location of substituents, flavonoids can be divided into 8 categories: flavones, flavanones, flavanols, isoflavones, flavonols, anthocyanidins, flavanonols, and chalcone. Results showed that the content of flavanones, flavanols, isoflavones, flavonols, and flavanonols in black/purple rice significantly increased by 1.81, 0.57, 3.25, 0.7 and 0.96 times, respectively after extrusion puffing. Isoflavones exhibits the great number differences between BR and EBR samples. Among these, isoflavones are known to have a positive effect on lowering low density lipoproteins cholesterol levels, which lead to a lower incidence of heart disease in people (31). Additionally, we also detected a kind of flavonoid: xanthone (mangiferin and isomangiferin). These compounds are structurally diverse secondary metabolites that exhibit antimicrobial, antidiabetic, and antitumor properties in pharmacological contexts (32). Some polyphenols and flavonoids in plants are usually combined with polysaccharides, which are unable to be extracted. Given that extrusion puffing can break down ester bonds in cell walls, proteins, certain flavonoids, and other macromolecular structures owing to its high temperature and pressure levels (8), potentially thereby releasing the bioactive compounds otherwise bound within black/purple rice. As a result, extrusion puffing contributes to increased flavonoid diversity within black/purple rice, improving the variety and content of beneficial metabolites in EBR samples.

The OPLS-DA model was further used to analyze differentially abundant metabolites in EBR and BR samples. Remarkably, the identified upregulated differentially abundant metabolites comprised a variety of pharmacologically active compounds including quercetin, hydroxygenkwanin, isorhamnetin, and (−)-catechin, which have been reported to exhibit anticancer, anti-inflammatory, antioxidant, and gut probiotic effects (33–36). These findings highlight the significant improvements in healthy flavonoid content in EBR samples as compared to BR samples. On the other hand, the majority of downregulated metabolites were anthocyanins. Given their high levels of instability, anthocyanins can be influenced by multiple factors including enzymatic activity, light, humidity, temperature, and pH (37). Therefore, the reduced anthocyanin levels in black/purple rice following extrusion could plausibly be attributed to high levels of pressure and temperature conditions to which these samples were subjected. Notably, levels of delphinidin-3-O-sophoroside and delphinidin-3-O-glucoside rose following extrusion treatment, whereas the amount of dihydromyricetin, which can serve as a delphinidin-3-O-sophoroside and delphinidin-3-O-glucoside precursor, declined significantly. Dihydromyricetin present in black/purple rice may thus be subjected to temperature-mediated induction of delphinidin-3-O-sophoroside and delphinidin-3-O-glucoside synthesis early during the extrusion puffing process. In summary, the qualitative and quantitative analyses of flavonoid metabolite content in EBR and BR samples suggested that while there may be some degradation of anthocyanin during processing, it is more conductive to enhancing the abundance and diversity of flavonoids.

### Extrusion puffing improved flavor and weakened unpleasant odor

Flavor and appearance are key determinants of food quality, and volatile aromatic compounds play a central role in shaping the flavor of a given food (38). Aromatic volatile compounds (VCs) are most often detected through gas chromatography–mass spectrometry (GC–MS) and gas chromatography-ion mobility spectrometry (GC-IMS) approaches, with the latter relying on a novel gas-phase separation approach and robust detection tools to rapidly detect even trace levels of compounds of interest (38). Here, a GC-IMS approach was utilized to detect flavor-related VCs in BR and EBR samples. The results showed that a total of 72 volatile components and 13 dimers were identified, including esters (15), aldehydes (18), ketones (9), alcohols (14), organic acids (2), heterocyclics (7), and other VCs (7), among which aldehydes comprised the largest overall proportion. In generally, alcohols and aldehydes are regarded as important aromatic compounds on account of their pleasant aromas and low threshold values (39). Lipids are critical precursors required for flavor establishment, given that free unsaturated fatty acids can undergo oxidation to form esters, aldehydes, and alcohols (40), with a concomitant drop in fat content. Meanwhile, pyrazine- and furan-containing heterocyclic compounds can be generated through the Maillard reaction of 1, 4-dideoxyketolose and glycine, optimizing food flavor with a nutty or toasty flavor that can be detected even at very low levels (41). Extrusion puffing can also result in the production of more complex ketones via the Maillard reaction (42). Furthermore, extrusion puffing treatment led to a marked reduction in the levels of numerous unfavorable volatile compounds. For instance, extrusion brought about a decline in the levels of the unpleasant-smelling 2-methylpropanoic acid, which is closely related to deterioration and decline in food quality (43). Decalin, an industrial solvent, has been reported to induce α_2u_-globulin nephropathy in male rats (44), and its levels in black/purple rice declined following extrusion puffing. As such, extrusion puffing can profoundly alter the VCs content in black/purple rice samples, likely imparting them with enhanced flavor given the observed formation of beneficial VCs and reductions in harmful VC levels following this form of processing.

In conclusion, the identification of fundamental nutritional compounds, differential flavonoid and volatile metabolites lays the groundwork for future research on the functional and nutritional value of various extrusion puffed products.

## Conclusion

Here, metabolomics profiling analyses of black/purple rice samples were performed to explore the impact of the extrusion puffing process on nutrient content and bioactive properties. Overall, extrusion puffed black/purple rice exhibited pronounced increases in protein, carbohydrate, and mineral content together with reductions in moisture and fat content. Additionally, extrusion puffing causes flavonoids and volatile compound content and variety rose markedly in black/purple rice samples. This suggests that extrusion puffing represents an effective means of substantially improving the functional and nutritional properties of black/purple rice, offering beneficial effects on consumer health. These data provide novel insights into the quality of extrusion puffed black/purple rice that will guide future efforts to establish how extrusion puffing can alter the nutrient content in a range of foods, thereby supporting the further development of a range of healthy food products.

## Data availability statement

The original contributions presented in the study are included in the article/Supplementary material, further inquiries can be directed to the corresponding authors.

## Author contributions

YM: investigation, formal analysis, writing – original draft, and review and editing. JL: investigation and formal analysis. YaX and YuX: writing – review and editing. CL and DS: supervision, project administration, and writing – review and editing. All authors contributed to the article and approved the submitted version.
