# Supplementary material for: Comprehensive improvement of nutrients and volatile compounds of black/purple rice by extrusion-puffing technology
Source: Front Nutr. 2023 Oct 11;10:1248501. doi: 10.3389/fnut.2023.1248501 (PMC10598597; doi:10.3389/fnut.2023.1248501)
Supplement: Supplementary file 1 [file Data_Sheet_1.docx]

Table S1 Composition of flavonoids in BR and EBR.

| Composition | BR (nmol/g) | EBR (nmol/g) | Composition | BR (nmol/g) | EBR (nmol/g) |
| --- | --- | --- | --- | --- | --- |
| Phlorizin | 0.3741±0.0587 | 0.3753±0.0178 | Isosilybin | 0.0157±0.0023 | 0.0154±0.0008 |
| Isoliquiritigenin | 0.0216±0.0029* | 0.0117±0.0016* | Taxifolin | 12.9106±1.9789 | 17.3586±0.5140* |
| 4,4'-Dimethoxychalcone | 0.0012±0.0004 | 0.0031±0.0006* | Dihydromyricetin | 1.1458±0.2654* | 0.2213±0.0468 |
| Naringenin chalcone | 0.5829±0.0456 | 1.0722±0.0800* | Astilbin | 0.1914±0.0552 | 0.4465±0.0785* |
| Trilobatin | 0.0544±0.0040* | 0.0335±0.0056 | Tectochrysin | 0.0022±0.0007 | 0.0012±0.0004 |
| Phloretin | 0.0162±0.0024* | 0.1311±0.0035 | Hydroxygenkwanin | ND | 0.0053±0.0010* |
| (-)-Catechin | 0.1818±0.0121 | 1.6821±0.1613* | 7,4'-Di-O-methylapigenin | 0.1172±0.0059* | 0.0658±0.0068 |
| (-)-Catechin gallate | 0.0535±0.0114 | 0.1515±0.0553* | Eupatorin | 0.0004±0.0001 | 0.0007±0.0001* |
| (-)-Epicatechin | 0.1063±0.0247* | ND | Cynaroside | 1.5332±0.0913* | 1.1689±0.1207 |
| (-)-Gallocatechin gallate | ND | 0.2444±0.0201* | Apigenin | 0.2407±0.0659 | 0.2475±0.0128 |
| (-)-Epigallocatechin | 1.2395±0.1029* | 0.4087±0.0111 | Luteolin | 0.9614±0.0945 | 1.1459±0.0288* |
| Liquiritin | 0.0070±0.0005 | 0.0325±0.0080* | Nicotiflorin | 0.0498±0.0073 | 0.0436±0.0011 |
| Eriodictyol | 2.8057±0.0431 | 8.5131±0.5645* | Oroxin A | 0.0833±0.0100 | 0.0839±0.0101 |
| Hesperidin | 0.2764±0.0733 | 0.2789±0.0189 | Sakuranetin | 0.0048±0.0006* | ND |
| Liquiritigenin | 0.0047±0.0006 | 0.0103±0.0002* | Cyanidin-3-O-glucoside | 108.4901±4.9959* | 63.8087±6.2447 |
| Pinocembrin | 0.0025±0.0016 | 0.0044±0.0010* | Cyanidin-3-O-arabinoside | 0.0007±0.0001* | ND |
| Eriocitrin | 0.0163±0.0041 | 0.0129±0.0005 | Cyanidin-3-O-xyloside | 0.2316±0.0319* | 0.0604±0.0144 |
| Narirutin | 0.0515±0.0036* | 0.0384±0.0053 | Cyanidin-3-O-rutinoside | 5.3590±0.7574* | 2.8578±0.3406 |
| Dihydrokaempferol | 0.1498±0.0153* | 0.1138±0.0094 | Cyanidin-3-O-sophoroside | 1.8333±0.0278* | 1.4990±0.0063 |

Table S1 (Continued)

| Composition | BR (nmol/g) | EBR (nmol/g) | Composition | BR (nmol/g) | EBR (nmol/g) |
| --- | --- | --- | --- | --- | --- |
| Cyanidin-3-O-(6-O-p-coumaroyl)-glucoside | 0.1168±0.0147* | 0.0627±0.0011 | Narcissin | 0.7110±0.0809* | 0.5103±0.0539 |
| Cyanidin-3-O-(6-O-malonyl-beta-D-glucoside) | 0.0485±0.0079* | 0.0128±0.0019 | Diosmetin | 1.0363±0.1606 | 1.1504±0.0940 |
| Delphinidin-3-O-sophoroside | 0.0017±0.0005 | 0.0197±0.0054* | Pedalitin | 0.0238±0.0020 | 0.0257±0.0058 |
| Delphinidin-3-O-glucoside | 0.0397±0.0064 | 0.1472±0.0269* | Quercimeritrin | 1.1804±0.3409 | 0.9764±0.0391 |
| Malvidin-3-O-glucoside | 0.0273±0.0021* | 0.0182±0.0005 | Isorhamnetin 3-O-glucoside | 5.7345±0.3252* | 4.7384±0.0782 |
| Malvidin-3-O-(6-O-p-coumaroyl)-glucoside | 0.0135±0.0042 | 0.0069±0 | Quercetin | 0.9186±0.0608 | 31.3081±2.1239* |
| Pelargonidin-3-O-sophoroside | 0.0064±0.0002* | 0.0052±0.0006 | Quercitrin | 0.0928±0.0079* | 0.0686±0.0022 |
| Pelargonidin-3-O-glucoside | 0.1740±0.0117* | 0.0724±0.0110 | Miquelianin | 0.0300±0.0041 | 0.0361±0.0068 |
| Scutellarin | 0.0633±0.0062 | 0.0645±0.0081 | Hyperoside | 36.6732±0.1944 | 32.2505±3.7593 |
| 7,4'-Dihydroxyflavone | 0.0226±0.0016* | 0.0167±0.0023 | Avicularin | 0.0536±0.0069 | 0.0435±0.0002 |
| Oroxin B | 0.0600±0.0065* | 0.0330±0.0029 | Isorhamnetin | 0.4603±0.0274 | 8.6829±0.3771* |
| Genkwanin | 0.0479±0.0092 | 0.0420±0.0043 | Kaempferol 3-neohesperidoside | 0.1424±0.0191* | 0.1004±0.0099 |
| Homoplantaginin | 0.2590±0.0338 | 0.2010±0.0042 | Myricetin | 0.0746±0.0029 | 0.1127±0.0103* |
| Diosmin | 1.5704±0.0679* | 1.2089±0.0410 | Quercetin 3-O-(6''-galloyl)-β-D-galactopyranoside | 0.0444±0.0050* | ND |
| Apigenin 7-glucoside | 0.0600±0.0082 | 0.0437±0.0060 | Tiliroside | 0.0048±0.0002* | ND |
| Kaempferol | ND | 0.1502±0.0268* | Kaempferitrin | ND | 0.0444±0.0143* |

Table S1 (Continued)

| Composition | BR (nmol/g) | EBR (nmol/g) | Composition | BR (nmol/g) | EBR (nmol/g) |
| --- | --- | --- | --- | --- | --- |
| Spiraeoside | 0.0998±0.0066 | 0.1196±0.0109 | Mangiferin | ND | 0.0205±0.0068* |
| Laricitrin | ND | 0.0801±0.0077* | Isomangiferin | ND | 0.0126±0.0029* |
| Afzelin | ND | 0.0041±0.0004* | Pelargonidin-3-O-(6-O-p-coumaroyl)-glucoside | 0.0025±0.0003 | 0.0042±0.0007* |
| 2''-O-Galloylhyperin | 0.1759±0.0037* | ND | Peonidin-3-O-(6-O-p-coumaroyl)-glucoside | 0.0779±0.0141 | 0.1095±0.0016* |
| Baimaside | 0.2402±0.0446 | 0.2182±0.0223 | Peonidin-3-O-rutinoside | 0.7373±0.0504* | 0.4636±0.0508 |
| Isorhamnetin-3-O-neohespeidoside | 0.1475±0.0095* | 0.1092±0.0034 | Peonidin-3-O-(6-O-malonyl-beta-D-glucoside) | 0.0853±0.0222* | 0.0175±0.0021 |
| Tectorigenin | 0.0028±0.0007 | 0.0070±0.0001* | Peonidin | 0.0052±0.0017* | ND |
| Daidzein | ND | 0.0221±0.0015* | Peonidin-3,5-O-diglucoside | 0.1399±0.0019 | 0.1565±0.0013* |
| Genistein | ND | 0.0621±0.0024* | Peonidin-3-O-glucoside | 10.3822±1.3414* | 5.0658±0.6389 |
| Daidzin | 0.0258±0.0010 | 0.0890±0.0083* | Petunidin-3-O-glucoside | 0.0204±0.0002 | 0.0308±0.0014* |
| Genistin | 0.1102±0.0327 | 0.4126±0.0351* | Rutin | 0.1942±0.0283 | 0.2559±0.0220* |

All data are averages of three measurements with standard deviation.

* And * indicate significant correlations at P < 0.05. ND, not detected.

Table S2 The signal intensity of 85 volatile components detected in BR and EBR.

| Compound | Signal intensity (a.u.) | | | Compound | | Signal intensity (a.u.) | | | |  |
| --- | --- | --- | --- | --- | --- | --- | --- | --- | --- | --- |
|  | BR | EBR | |  | | BR | | EBR | |  |
| Nonanal-monomer | 2625.04±176.48 | 2242.88±72.08 | | Hexanal-monomer | | 874.00±96.49 | | 1397.58±9.08 | |  |
| Nonanal-dimer | 876.65±107.94 | 597.88±45.41 | | Hexanal-dimer | | 342.79±14.07 | | 319.47±21.13 | |  |
| Carvone | 239.54±20.13 | 584.03±32.36 | | (E)-2-Hexenal | | 235.09±58.91 | | 198.19±8.80 | |  |
| Octanal | 480.70±23.78 | 199.92±13.50 | | Compound from HS-Vial Septum | | 349.96±4.22 | | 601.88±25.38 | |  |
| Ethyl hexanoate | 118.73±8.06 | 272.02±63.70 | | Allyl isothiocyanate | | 95.85±15.95 | | 434.70±25.83 | |  |
| 2-Furancarboxylic acid,  methyl ester-monomer | 453.26±57.27 | 1093.83±140.69 | | 2,3-Butanediol | | 619.33±13.93 | | 633.49±7.65 | |  |
| 2-Furancarboxylic acid,  methyl ester-dimer | 58.94±8.51 | 253.36±33.68 | | Ethyl butyrate | | 166.52±25.23 | | 484.29±34.57 | |  |
| (E)-Hept-2-enal-monomer | 187.91±10.19 | 378.87±12.01 | | 2-Propanone, 1-(acetyloxy)- | | 701.69±23.71 | | 822.08±25.46 | |  |
| (E)-Hept-2-enal0dimer | 33.39±1.99 | 101.61±22.02 | | 3-Methylthiopropanal | | 357.08±10.86 | | 156.46±10.74 | |  |
| 3-(Methylsulfanyl) propanol | 276.82±14.87 | 270.19±10.05 | | 3-Methylbutan-1-ol-dimer | | 1049.83±38.82 | | 82.61±8.57 | |  |
| Propyl butanoate-monomer | 394.54±2.71 | 428.38±5.06 | | 3-Methylbutan-1-ol-monomer | | 457.96±21.40 | | 122.37±13.84 | |  |
| Propyl butanoate-dimer | 1796.86±81.26 | 4049.74±174.56 | | 2-Hexanone | | 325.88±22.84 | | 117.70±3.21 | |  |
| Methyl hexanoate-dimer | 129.78±3.48 | 516.51±29.26 | | 2-Methylpropanoic acid | | 640.94±47.73 | | 156.03±5.35 | |  |
| Methyl hexanoate-monomer | 384.43±33.01 | 1598.07±182.12 | | Diethoxy-1,1-ethane-dimer | | 608.00±44.40 | | 502.21±12.29 | |  |
| Heptanal-monomer | 265.87±42.75 | | 305.65±85.00 | | Diethoxy-1,1-ethane-monomer | | 924.11±51.89 | | 550.19±15.18 | |
| Heptanal-dimer | 628.11±24.34 | | 518.53±49.77 | | 4-Methyl-2-pentanol | | 167.87±10.36 | | 341.46±16.79 | |
| 2,6-Dimethylpyrazine | 163.08±2.77 | | 421.38±26.32 | | 3-Heptanol | | 394.17±14.06 | | 108.27±3.90 | |

Table S2 (Continued)

| Compound | Signal intensity (a.u.) | | Compound | Signal intensity (a.u.) | |
| --- | --- | --- | --- | --- | --- |
|  | BR | EBR |  | BR | EBR |
| 2-Methyl-3-furanthiol | 457.15±57.86 | 847.98±60.47 | 2(3H)-Furanone, dihydro-5-methyl- | 133.48±6.25 | 132.63±7.70 |
| 2-Acetylfuran | 143.51±20.42 | 286.95±9.31 | 2-Methylpropyl butanoate | 36.18±1.45 | 55.07±14.15 |
| Acetic acid butyl ester-monomer | 275.47±15.05 | 2354.35±127.04 | Mesityl oxide | 231.33±13.80 | 262.26±6.62 |
| Acetic acid butyl ester-dimer | 268.33±6.63 | 548.92±52.37 | 4-Methylpentanol | 55.69±15.85 | 67.50±5.71 |
| Formic acid, 3-methylbutyl ester | 347.52±25.86 | 212.08±18.73 | 3-Buten-1-ol, 3-methyl- | 101.10±23.04 | 299.13±23.69 |
| Ethyl 2-methylbutyrate | 11.62±2.52 | 11.71±3.21 | 1-Butanol | 1613.92±181.72 | 1449.39±21.17 |
| 2-Butoxyethanol | 32.28±1.29 | 18.65±2.72 | Pentanal | 416.86±21.13 | 428.62±70.39 |
| Pentanoic acid | 37.80±1.76 | 47.59±4.48 | 1,4-Dioxane | 3206.00±178.87 | 5320.55±267.78 |
| 2-n-Butylfuran | 29.02±4.04 | 42.13±3.32 | Acetic acid ethyl ester-monomer | 154.03±17.86 | 161.59±2.26 |
| 2-Methyl-2-pentenal | 51.30±6.88 | 51.38±4.63 | Acetic acid ethyl ester-dimer | 117.91±13.40 | 73.76±5.45 |
| Methyl 3-methylbutanoate | 79.51±4.83 | 129.92±0.57 | Butanal | 283.89±66.34 | 433.87±29.48 |
| 3-Methyl-2-butenal | 170.89±14.62 | 116.01±3.96 | 2-Butanone-dimer | 252.97±92.87 | 774.15±71.91 |
| (E)-2-Hexen-1-ol | 62.65±31.51 | 48.25±4.76 | 2-Butanone-monomer | 615.09±59.76 | 910.59±45.58 |
| Isoamyl acetate | 85.39±23.86 | 77.05±9.18 | Propanal | 9524.16±369.55 | 9543.73±502.65 |
| 1-Octen-3-one | 100.97±11.67 | 217.87±31.76 | Methanethiol | 1799.68±53.14 | 1174.62±45.96 |
| Isoamyl butyrate | 111.43±8.61 | 221.45±15.46 | Ethanol | 1314.00±59.73 | 1724.72±307.14 |
| Methyl-5-hepten-2-one | 473.12±22.82 | 412.80±7.35 | 2-Propenal, 2-methyl- | 1314.09±97.45 | 867.01±53.05 |
| 3-Octanone | 143.18±9.71 | 71.10±12.23 | Ethylene glycol dimethyl ether | 808.27±45.36 | 691.92±13.97 |
| 2-Pentyl furan | 140.02±16.44 | 122.00±5.59 | 2-Methyl-1-propanol | 412.93±17.77 | 67.74±5.18 |

Table S2 (Continued)

| Compound | Signal intensity (a.u.) | | Compound | Signal intensity (a.u.) | |
| --- | --- | --- | --- | --- | --- |
|  | BR | EBR |  | BR | EBR |
| 2-Octanol | 128.01±19.13 | 137.73±42.78 | 3-Methylbutanal | 769.59±77.12 | 646.44±90.09 |
| Octamethylcyclotetrasiloxane | 121.37±7.06 | 153.44±10.28 | 2,3-Butanedione | 329.80±26.33 | 235.15±17.51 |
| (E)-2-Octenal | 98.94±5.92 | 103.39±4.28 | Ethyl acrylate | 56.83±7.16 | 182.32±18.57 |
| Benzene acetaldehyde | 83.61±5.51 | 56.42±4.78 | 3-Butenenitrile | 181.73±21.5 | 152.34±9.25 |
| Decalin | 87.88±7.37 | 68.56±5.18 | Isopropyl alcohol | 462.59±10.35 | 250.18±21.27 |
| Limonene-monomer | 77.82±5.02 | 61.38±6.42 | (Z)-4-Heptenal | 210.41±8.87 | 151.11±3.04 |
| Limonene-dimer | 48.20±4.11 | 63.35±10.82 |  |  |  |

All data are averages of three measurements with standard deviation.
