# Supplementary figures and images for: Comprehensive improvement of nutrients and volatile compounds of black/purple rice by extrusion-puffing technology
Source: Front Nutr. 2023 Oct 11;10:1248501. doi: 10.3389/fnut.2023.1248501 (PMC10598597; doi:10.3389/fnut.2023.1248501)

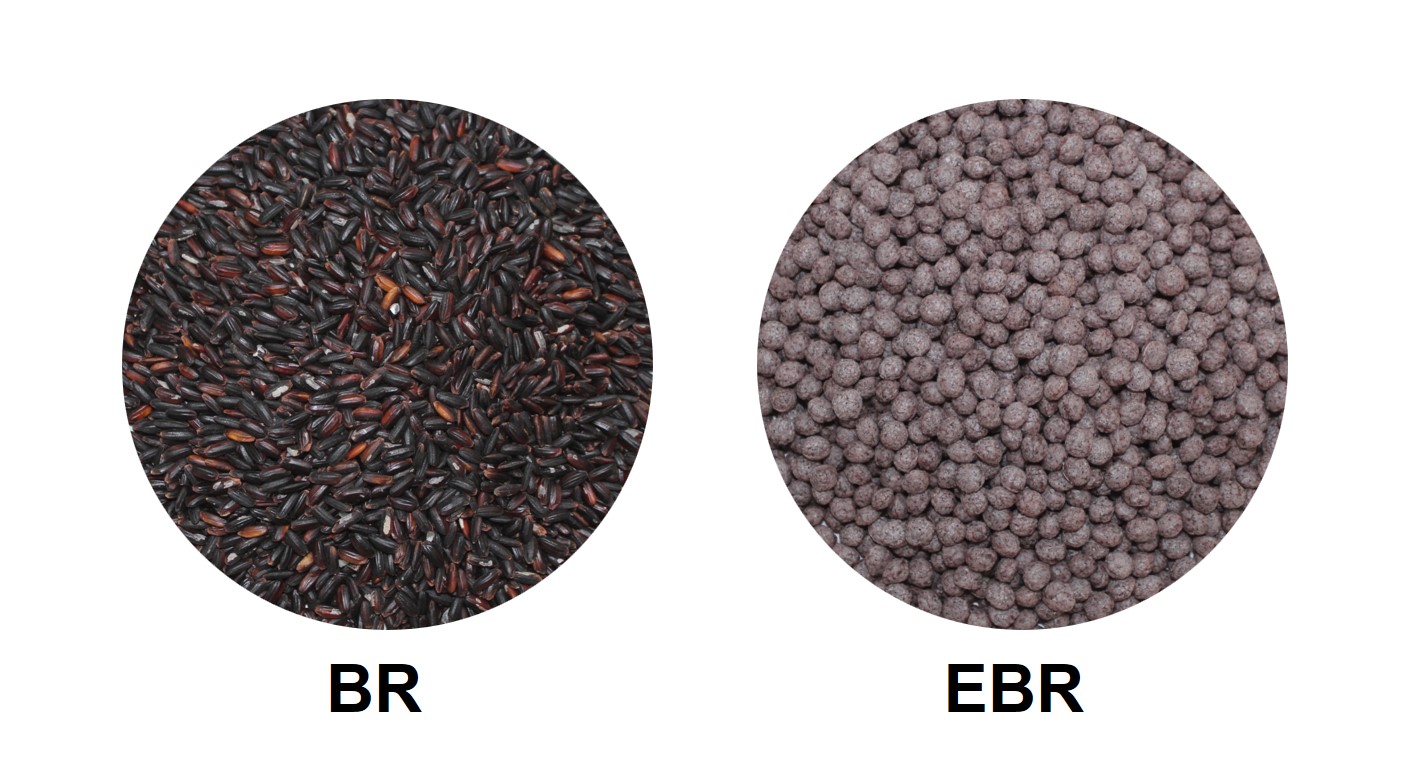

Supplement: Supplementary file 2 [file Image_1.JPEG]
